# Intraoperative intact parathyroid hormone monitoring and frozen section diagnosis are essential for successful parathyroidectomy in secondary hyperparathyroidism

**DOI:** 10.3389/fmed.2022.1007887

**Published:** 2022-11-07

**Authors:** Takahisa Hiramitsu, Yuki Hasegawa, Kenta Futamura, Manabu Okada, Norihiko Goto, Shunji Narumi, Yoshihiko Watarai, Yoshihiro Tominaga, Toshihiro Ichimori

**Affiliations:** Department of Transplant and Endocrine Surgery, Japanese Red Cross Aichi Medical Center Nagoya Daini Hospital, Nagoya, Japan

**Keywords:** secondary hyperparathyroidism, parathyroidectomy, intraoperative intact PTH monitoring, frozen section, imaging diagnosis

## Abstract

**Background:**

Total parathyroidectomy (PTx) is often performed to treat secondary hyperparathyroidism (SHPT). Successful PTx is essential to prevent recurrent and persistent SHPT because remnant parathyroid glands (PTGs) in the neck can be stimulated and may secrete excessive parathyroid hormone (PTH) in end-stage renal disease. However, to date, few studies have investigated factors contributing to successful PTx before the completion of surgery.

**Materials and methods:**

Between August 2010 and February 2020, 344 patients underwent total PTx, transcervical thymectomy, and forearm autograft for SHPT at our institute. Factors contributing to successful PTx before the completion of surgery were investigated. Preoperative imaging diagnoses, including computed tomography, ultrasonography, technetium-99m methoxyisobutylisonitrile (^99m^Tc-MIBI) scintigraphy, intraoperative intact PTH (IOIPTH) monitoring, and frozen section histologic diagnosis, were performed. Successful PTx was defined as intact PTH level < 60 pg/mL on postoperative day 1. A sufficient decrease in IOIPTH level was defined as > 70% decrease in intact PTH levels measured 10 min after total PTx and transcervical thymectomy compared to intact PTH levels measured before skin incision. Logistic regression analysis was conducted to investigate factors contributing to PTx success.

**Results:**

Univariate analysis showed that the number of all PTGs identified preoperatively by imaging modalities and the specimens submitted for frozen section diagnosis, which surgeon presumed to be PTGs, were not significant factors contributing to successful PTx. However, multivariate analysis revealed that the number of PTGs identified by frozen section diagnosis (*P* < 0.001, odds ratio [OR] 4.356, 95% confidence interval [CI] 2.499–7.592) and sufficient decrease in IOIPTH levels (*P* = 0.001, OR 7.847, 95% CI 2.443–25.204) significantly contributed to successful PTx.

**Conclusion:**

Sufficient intact PTH level decrease observed on IOIPTH monitoring and the number of PTGs identified by frozen section diagnosis contributed to successful PTx for SHPT. IOIPTH monitoring and frozen section diagnosis are essential for achieving successful PTx for SHPT.

## Introduction

Secondary hyperparathyroidism (SHPT) is often observed in patients with end-stage renal disease (ESRD) and can result in excessive parathyroid hormone (PTH) secretion, leading to osteodystrophy, bone fractures, and increased risks of mortality and cardiovascular events (1–4). SHPT is usually diagnosed by the serum PTH levels and imaging studies, including computed tomography (CT), ultrasonography (US), and technetium-99m methoxyisobutylisonitrile (^99m^Tc-MIBI) scintigraphy. Before the development of calcimimetics, vitamin D receptor activators such as calcitriol, paricalcitol, and supplemental calcium were administered to treat SHPT. The effect of these medications was limited to control SHPT. The necessity of treating SHPT due to the previously mentioned adverse effects is widely recognized. As a result, the number of parathyroidectomies (PTx) increased (5). After the development of calcimimetics, the administration of calcimimetics can effectively control SHPT and reduce the volume of parathyroid glands (PTGs) (6–9). The number of PTx decreased dramatically (5, 9). Although calcimimetics are usually administered to treat SHPT, PTx is required in patients who do not respond to drug treatment and those who cannot tolerate calcimimetics because of drug allergies and adverse effects (9–13). Total PTx, transcervical thymectomy, and forearm autograft are frequently performed (1, 14). In PTx, complete removal of the PTGs is essential to prevent recurrent and persistent SHPT in the neck because remnant PTGs are continuously stimulated under ESRD and readily secrete excess PTH (15, 16). In patients with recurrent and persistent SHPT in the neck, additional PTx is required. However, additional PTx may result in injury to the recurrent laryngeal nerve due to adhesions after the initial PTx. Preoperative and intraoperative factors, such as preoperative imaging studies, intraoperative intact PTH (IOIPTH) monitoring, and frozen section diagnosis, may determine the success of PTx before the completion of surgery. Several separate reports have suggested the usefulness of preoperative imaging studies and IOIPTH monitoring for successful PTx; nonetheless, no reports have comprehensively investigated the impact of these factors on the success of PTx for SHPT (17–24). Thus, in this study, we comprehensively investigated the factors contributing to successful PTx before the completion of surgery.

## Materials and methods

### Study design

This retrospective cohort study was approved by the Institutional Review Board of the Japanese Red Cross Aichi Medical Center Nagoya Daini Hospital (approval number 1547; Aichi, Japan). The factors associated with successful PTx for SHPT before the completion of surgery were investigated. This study was conducted according to the principles of the Declaration of Helsinki and Strengthening the Reporting of Observational Studies in Epidemiology (STROBE) guidelines.

### Participants

Between August 2010 and February 2020, consecutive patients who underwent total PTx, transcervical thymectomy, and forearm autograft for SHPT at our institute were included in this study. All patients were included in this study. The patients were followed up until March 2022. Patients were followed up to assess recurrent and persistent SHPT at 1, 3, 6, and 12 months and annually after PTx. All patient data were collected retrospectively from medical records and analyzed anonymously; therefore, obtaining informed consent from the participants was not required.

### Indication of parathyroidectomy for secondary hyperparathyroidism and preoperative evaluation

Parathyroidectomy for SHPT is indicated based on the chronic kidney disease-related mineral and bone disorder (CKD-MBD) guidelines in Japan (10). Patients who were refractory to drug treatment (intact PTH levels ≥ 500 pg/mL) and could not take calcimimetics were indicated for PTx. PTGs were evaluated by radiologists using imaging studies such as CT, US, and ^99m^Tc-MIBI scintigraphy. US was performed by laboratory technicians who were approved as specialists by The Japanese Association of Breast and Thyroid Sonology.

### Operative procedure

Parathyroidectomy, transcervical thymectomy, and forearm autograft were performed in all patients included in this study. All operations were performed or supervised by experienced surgeons who had performed at least 200 parathyroidectomies for SHPT. Frozen section diagnosis and IOIPTH monitoring were performed in all patients. Intact PTH levels were measured by Elecsys PTH STAT immunoassay (Roche DIAGNOSTICS, Tokyo, Japan) and ST AIA-PACK Intact PTH (Tosoh Corporation, Tokyo, Japan). Intact PTH levels were measured before skin incision and 10 min after total PTx and transcervical thymectomy. A decrease in intact PTH levels > 70% was defined as a sufficient decrease in IOIPTH levels for successful PTx (22). Successful PTx was defined as intact PTH levels < 60 pg/mL on postoperative day (POD) 1, which was shown as the lower range of the targeted intact PTH levels (60–240 pg/mL) in the CKD-MBD guidelines in Japan (10).

### Frozen section and paraffin section histologic diagnoses

Pathologists evaluated frozen and paraffin sections. A small amount of resected tissues was submitted for frozen section diagnosis. The samples submitted for frozen section diagnosis were also embedded in paraffin wax for paraffin section diagnosis. A final paraffin section diagnosis was performed for the resected samples as a part of the final diagnosis.

### Statistical analysis

Categorical variables were analyzed using the Chi-square test or Fisher’s exact test, whereas continuous variables were analyzed using the Kruskal–Wallis test. To compare the incidence of recurrent and persistent SHPT between successful and unsuccessful PTx groups, Cox regression analysis adjusted with inverse probability of treatment weighting (IPTW) was performed, which included all of the following covariates: sex, age, body mass index (BMI), dialysis vintage, and intact PTH levels at admission. To examine the factors contributing to successful PTx before the completion of the surgery, the logistic regression models used included all of the following covariates: sex, age, height, body weight, and BMI; dialysis vintage; preoperative serum calcium levels corrected for serum albumin level; preoperative serum phosphorus levels; number of PTGs identified by preoperative CT; number of PTGs identified by preoperative US; number of PTGs identified by preoperative ^99m^Tc-MIBI; number of PTGs identified by preoperative CT, US, and ^99m^Tc-MIBI; number of samples submitted for frozen section diagnosis; number of PTGs identified by frozen section diagnosis; intact PTH levels at admission; and decrease in IOIPTH levels > 70%. In the multivariate logistic regression analysis, covariates of which *P*-value was < 0.05 in the univariate logistic regression analysis were used. In the subgroup analysis of patients in whom < 4 PTGs were identified by frozen section diagnosis during surgery, logistic regression models were used to examine factors contributing to successful PTx before the completion of the surgery, and all of the following covariates were included: sex, age, height, body weight, and BMI; dialysis vintage; preoperative serum calcium levels corrected for serum albumin level; preoperative serum phosphorus level; number of PTGs identified by preoperative CT; number of PTGs identified by preoperative US; number of PTGs identified by preoperative ^99m^Tc-MIBI; number of PTGs identified by preoperative CT, US, and ^99m^Tc-MIBI; intact PTH levels at admission, and decrease in IOIPTH levels > 70%. Statistical analyses were performed using IBM SPSS^®^ Statistics for Windows (version 23.0; IBM Corp., Armonk, NY, USA) and R version 4.0.2 (R Core Team 2020), with statistical significance set at *P*-value < 0.05 for all analyses.

## Results

### Study population

**FIGURE 1:**
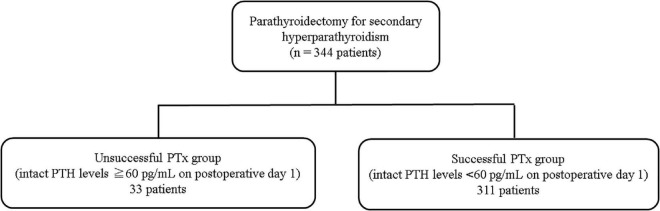
Patient flow chart.

A total of 344 PTx procedures were performed between August 2010 and February 2020. All patients were followed between August 2010 and March 2022 (median observation period: 26.5 [interquartile range, 11.0–46.5] months) and were included in the final analysis. Patients were classified into the following two groups based on intact PTH levels on POD 1: the unsuccessful PTx group (≥60 pg/mL) and the successful PTx group (<60 pg/mL) (Figure 1).

### Patient characteristics

Regarding patient characteristics, no significant differences were identified between the two groups, except for that in dialysis vintage (*P* = 0.031) (Table 1).

**TABLE 1:** Patient characteristics.

	Unsuccessful PTx group	Successful PTx group	*P-value*	Odds ratio	95% CI	
	*n* = 33	*n* = 311				
Sex male (%)	17 (51.5)	173 (55.6)	0.714	1.180	0.575	2.420
Age years (SD)	55.4 (10.2)	55.9 (12.2)	0.589			
Height cm (SD)	159.3 (9.7)	161.2 (9.5)	0.153			
Body weight kg (SD)	56.2 (15.1)	59.5 (14.8)	0.057			
Body mass index kg/m^2^ (SD)	22.0 (4.5)	22.7 (4.2)	0.126			
Dialysis vintage months (SD)	163.7 (81.0)	133.6 (78.5)	**0.031**			
Preoperative serum albumin level g/dL (SD)	3.98 (0.35)	3.93 (0.42)	0.606			
Preoperative serum alkaline phosphatase level U/L (SD)	380.3 (212.8)	419.0 (459.4)	0.620			
Preoperative serum calcium level mg/dL (SD)	9.6 (0.8)	9.6 (0.8)	0.643			
Preoperative serum calcium level corrected by serum albumin level mg/dL (SD)	9.7 (0.8)	9.8 (0.8)	0.482			
Preoperative serum phosphorus level mg/dL (SD)	5.9 (1.3)	5.9 (1.6)	0.671			
Preoperative CT (%)	33 (100.0)	311 (100.0)	NA			
Preoperative US (%)	33 (100.0)	310 (99.7)	>0.999			
Preoperative MIBI (%)	32 (97.0)	304 (97.7)	0.558	1.357	0.162	11.383
Number of PTGs identified by preoperative CT (SD)	2.3 (1.1)	2.3 (1.1)	>0.999			
Number of PTGs identified by preoperative US (SD)	1.8 (1.2)	2.2 (1.1)	0.868			
Number of PTGs identified by preoperative ^99m^Tc-MIBI (SD)	1.7 (1.0)	1.6 (1.0)	0.499			
Number of PTGs identified by preoperative CT, US, and ^99m^Tc-MIBI (SD)	2.8 (1.1)	2.9 (1.0)	0.811			
Intact PTH level at admission pg/mL (SD)	875.5 (793.2)	696.4 (603.7)	0.069			

CI, confidence interval; CT, computed tomography; PTGs, parathyroid glands; PTH, parathyroid hormone; SD, standard deviation; ^99^*^m^*Tc-MIBI, technetium-99m methoxyisobutylisonitrile scintigraphy; US, ultrasonography. Boldface indicates statistically significant results.

### Intraoperative results

Intraoperative results indicated significant differences between the unsuccessful and successful PTx groups in terms of the number of samples submitted for frozen section diagnosis (*P* = 0.004), number of PTGs identified by frozen section diagnosis (*P* < 0.001), IOIPTH levels before skin incision (*P* = 0.023), IOIPTH levels 10 min after total PTx and transcervical thymectomy (*P* < 0.001), decrease in IOIPTH levels (*P* < 0.001), and decrease in IOIPTH levels > 70% (*P* < 0.001) (Table 2).

**TABLE 2:** Operative results.

		Unsuccessful PTx group	Successful PTx group	*P-value*	Odds ratio	95% CI	
		*n* = 33	*n* = 311				
Intraoperative results	Number of samples submitted for frozen section diagnosis (SD)	4.1 (1.4)	4.4 (0.8)	**0.004**			
	Number of PTGs identified by frozen section diagnosis (SD)	3.1 (0.9)	4.0 (0.5)	**< 0.001**			
	Intraoperative intact PTH levels before skin incision pg/mL (SD)	890.4 (703.9)	697.3 (559.5)	**0.023**			
	Intraoperative intact PTH levels 10 min after total PTx and transcervical thymectomy pg/mL (SD)	240.7 (207.6)	71.0 (57.2)	**< 0.001**			
	Decrease in intraoperative intact PTH levels% (SD)	67.4 (29.7)	87.9 (8.6)	**< 0.001**			
	Decrease in intraoperative intact PTH levels > 70% (%)	19 (57.6)	303 (97.4)	**< 0.001**	27.908	10.426	74.702
Postoperative results	Number of PTGs confirmed by paraffin section diagnosis of frozen specimens (SD)	3.0 (0.8)	4.0 (0.5)	**< 0.001**			
	Number of PTGs identified by paraffin section diagnosis (SD)	3.2 (1.0)	4.2 (0.6)	**< 0.001**			
	Rate of concordant PTGs by imaging diagnosis using CT% (SD)	60.2 (30.5)	53.8 (26.0)	0.129			
	Rate of concordant PTGs by imaging diagnosis using US% (SD)	60.2 (30.9)	52.5 (26.2)	0.103			
	Rate of concordant PTGs by imaging diagnosis using ^99m^Tc-MIBI% (SD)	44.1 (34.0)	36.5 (24.0)	0.216			
	Rate of concordant PTGs by imaging diagnosis using CT, US, and ^99m^Tc-MIBI% (SD)	74.0 (26.6)	67.9 (25.2)	0.181			
	Intact PTH levels on POD 1 pg/mL (SD)	178.3 (137.4)	10.0 (11.5)	**< 0.001**			
	Persistent and recurrent SHPT in the neck or mediastinum during the observation period %	5 (15.2)	5 (1.6)	**0.001**	0.092	0.025	0.335
	Observation period months (SD)	47.8 (28.4)	31.1 (27.0)	**0.001**			

CI, confidence interval; CT, computed tomography; POD, postoperative day; PTGs, parathyroid glands; PTH, parathyroid hormone; PTx, parathyroidectomy; SD, standard deviation; SHPT, secondary hyperparathyroidism; ^99*m*^Tc-MIBI, technetium-99m methoxyisobutylisonitrile scintigraphy; US, ultrasonography. Boldface indicates statistically significant results.

With respect to postoperative outcomes, significant differences were found between the unsuccessful and successful PTx groups in terms of the number of PTGs confirmed by paraffin section diagnosis for frozen samples (*P* < 0.001); number of PTGs identified by paraffin section diagnosis (*P* < 0.001); intact PTH levels on POD 1 (*P* < 0.001); persistent and recurrent SHPT in the neck or mediastinum during the observation period (*P* = 0.001); and the observation period (*P* = 0.001) (Table 2).

### Recurrent and persistent secondary hyperparathyroidism in successful and unsuccessful parathyroidectomy groups

**FIGURE 2:**
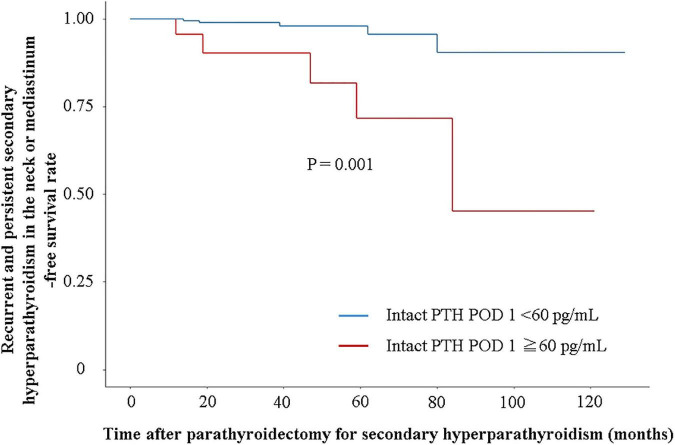
Recurrent and persistent secondary hyperparathyroidism-free survival rate in the neck or mediastinum. Recurrent and persistent secondary hyperparathyroidism-free survival rate is evaluated using Cox regression analysis adjusted with inverse probability of treatment weighting by sex, age, body mass index, dialysis vintage, and intact PTH levels at admission.

A total of 10 patients with recurrent and persistent SHPT (five patients in the successful PTx group and five patients in the unsuccessful PTx group) were identified (Table 3). The IPTW-adjusted Cox regression analysis showed a significantly lower incidence of recurrent and persistent SHPT in the successful PTx group (*P* = 0.001, hazard ratio [HR] 0.133, 95% confidence interval [CI] 0.039–0.453) (Figure 2).

**TABLE 3:** Characteristics of patients with recurrent and persistent secondary hyperparathyroidism.

Patient number	Localization of the parathyroid glands at additional PTx	Intact PTH on POD 1 (pg/mL) at initial PTx	Preoperative imaging diagnosis at initial PTx				Decrease in intact PTH levels on intraoperative monitoring at initial PTx (%)	Number of resected PTGs confirmed by final paraffin section diagnosis at initial PTx
					
			CT	US	^99m^Tc-MIBI	CT, US, or ^99m^Tc-MIBI		
1	Right lower PTG	42	Unidentified	Unidentified	Unidentified	Unidentified	87.7	4
2	Intrathymus	43	**Identified**	Unidentified	**Identified**	**Identified**	80.1	**3**
3	Mediastinum	36	Unidentified	Unidentified	Unidentified	Unidentified	82.3	4
4	Right upper PTG	59	**Identified**	**Identified**	**Identified**	**Identified**	82.7	4
5	Left lower PTG	19	Unidentified	Unidentified	Unidentified	Unidentified	81.5	4
6	Left lower PTG	**80**	Unidentified	Unidentified	Unidentified	Unidentified	76.1	**3**
7	Right upper PTG	**141**	Unidentified	Unidentified	Unidentified	Unidentified	85.8	**3**
8	Right lower PTG	**93**	Unidentified	**Identified**	Unidentified	**Identified**	**66.8**	**2**
9	Left lower PTG	**339**	**Identified**	**Identified**	**Identified**	**Identified**	**58.1**	**3**
10	Left lower PTG	**216**	Unidentified	**Identified**	Unidentified	**Identified**	**68.6**	4

CT, computed tomography; US, ultrasonography; PTH, parathyroid hormone; PTG, parathyroid gland; PTx, parathyroidectomy; ^99*m*^Tc-MIBI, technetium-99m methoxyisobutylisonitrile scintigraphy; POD, postoperative day. Boldface indicates statistically significant results.

### Logistic regression analysis for successful parathyroidectomy

Univariate logistic regression analysis revealed significant differences in dialysis vintage [*P* = 0.027, odds ratio (OR) 0.995, 95% CI 0.991–0.999], number of PTGs identified by frozen section diagnosis (*P* < 0.001, OR 6.927, 95% CI 3.799–12.631), and decrease in IOIPTH levels > 70% (*P* < 0.001, OR 27.908, 95% CI 10.426–74.702). However, no significant differences were observed in the number of samples submitted for frozen section diagnosis (*P* = 0.086, OR 1.626, 95% CI 0.934–2.829) and number of PTGs identified by preoperative CT (*P* = 0.807, OR 1.042, 95% CI 0.747–1.454), US (*P* = 0.920, OR 0.983, 95% CI 0.706–1.370), ^99m^Tc-MIBI (*P* = 0.540, OR 0.892, 95% CI 0.620–1.284), and a combination of CT, US, and ^99m^Tc-MIBI (*P* = 0.805, OR 1.044, 95% CI 0.741–1.472) (Table 4).

**TABLE 4:** Univariate logistic regression analysis for successful parathyroidectomy.

	*P-value*	Odds ratio	95% CI	
Sex (vs. female)	0.652	1.180	0.575	2.420
Age (years)	0.669	1.006	0.977	1.037
Height (cm)	0.153	1.028	0.990	1.068
Body weight (kg)	0.154	1.021	0.992	1.050
Body mass index (kg/m^2^)	0.295	1.052	0.957	1.156
Dialysis vintage (months)	**0.027**	0.995	0.991	0.999
Preoperative serum calcium level corrected by serum albumin level (mg/dL)	0.523	1.156	0.741	1.805
Preoperative serum phosphorus level (mg/dL)	0.860	0.980	0.786	1.222
Number of PTGs identified by preoperative CT	0.807	1.042	0.747	1.454
Number of PTGs identified by preoperative US	0.920	0.983	0.706	1.370
Number of PTGs identified by preoperative ^99m^Tc-MIBI	0.540	0.892	0.620	1.284
Number of PTGs identified by preoperative CT, US, and ^99m^Tc-MIBI	0.805	1.044	0.741	1.472
Number of samples submitted for frozen section diagnosis	0.086	1.626	0.934	2.829
Number of PTGs identified by frozen section diagnosis	**< 0.001**	6.927	3.799	12.631
Intact PTH levels at admission (pg/mL)	0.100	1.000	0.999	1.000
Decrease in intraoperative intact PTH levels > 70% (vs. decrease in intraoperative intact PTH levels ≤ 70%)	<**0.001**	27.908	10.426	74.702

CI, confidence interval; CT, computed tomography; PTGs, parathyroid glands; PTH, parathyroid hormone; SHPT, secondary hyperparathyroidism; ^99*m*^Tc-MIBI, technetium-99m methoxyisobutylisonitrile scintigraphy; US, ultrasonography. Boldface indicates statistically significant results.

Multivariate logistic regression analysis revealed significant differences in the number of PTGs identified by frozen section diagnosis (*P* < 0.001, OR 6.335, 95% CI 3.302–12.156) and decrease in IOIPTH levels > 70% (*P* = 0.001, OR 23.953, 95% CI 7.721–74.309) (Table 5).

**TABLE 5:** Multivariate logistic regression analysis for successful parathyroidectomy.

	*P-value*	Odds ratio	95% CI	
Dialysis vintage (months)	0.372	0.998	0.992	1.003
Number of PTGs identified by frozen section diagnosis	**< 0.001**	6.335	3.302	12.156
Decrease in intraoperative intact PTH levels > 70% (vs. decrease in intraoperative intact PTH levels ≤ 70%)	**0.001**	23.953	7.721	74.309

CI, confidence interval; PTGs, parathyroid glands; PTH, parathyroid hormone. Boldface indicates statistically significant results.

### Subgroup analysis of patients with <4parathyroid glands identified by frozen section diagnosis performed during surgery

Among 50 patients with <4 PTGs identified by frozen section diagnosis during surgery, 20 were part of the unsuccessful PTx group, whereas 30 were part of the successful PTx group. With regard to patient characteristics, the two groups showed significant differences in intact PTH levels at admission (*P* = 0.023), number of PTGs identified by frozen section diagnosis (*P* = 0.025), number of PTGs identified by final paraffin section diagnosis (*P* < 0.001), IOIPTH levels 10 min after total PTx and transcervical thymectomy (*P* < 0.001), decrease in IOIPTH levels (*P* < 0.001), decrease in IOIPTH levels > 70% (*P* = 0.001), and intact PTH levels on POD 1 (*P* < 0.001) (Supplementary Table 1).

Univariate logistic regression analysis for successful PTx revealed significant differences in decrease in IOIPTH levels > 70% (*P* = 0.004, OR 23.727, 95% CI 2.684–209.782) (Table 6).

**TABLE 6:** Univariate logistic regression analysis for successful parathyroidectomy in patients with < 4 parathyroid glands identified by frozen section diagnosis.

	*P-value*	Odds ratio	95% CI	
Sex (vs. female)	0.626	0.740	0.221	2.484
Age (years)	0.930	1.002	0.953	1.054
Height (cm)	0.302	1.032	0.972	1.096
Body weight (kg)	0.436	1.017	0.974	1.062
Body mass index (kg/m^2^)	0.790	1.020	0.880	1.183
Dialysis vintage (months)	0.303	0.996	0.990	1.003
Preoperative serum calcium level corrected by serum albumin level (mg/dL)	0.310	1.528	0.674	3.461
Preoperative serum phosphorus level (mg/dL)	0.476	0.875	0.605	1.264
Number of PTGs identified by preoperative CT	0.226	0.693	0.382	1.255
Number of PTGs identified by preoperative US	0.834	0.947	0.567	1.581
Number of PTGs identified by preoperative ^99m^Tc-MIBI	0.147	0.601	0.302	1.196
Number of PTGs identified by preoperative CT, US, and ^99m^Tc-MIBI	0.135	0.651	0.372	1.142
Number of samples submitted for frozen section diagnosis	0.561	1.163	0.699	1.937
Intact PTH levels at admission (pg/mL)	0.054	0.999	0.997	1.000
Decrease in intraoperative intact PTH levels > 70% (vs. decrease in intraoperative intact PTH levels ≤ 70%)	**0.004**	23.727	2.684	209.782

CI, confidence interval; CT, computed tomography; PTGs, parathyroid glands; PTH, parathyroid hormone; ^99*m*^Tc-MIBI, technetium-99m methoxyisobutylisonitrile scintigraphy; US, ultrasonography. Boldface indicates statistically significant results.

### Diagnosis of parathyroid glands by frozen section diagnosis and surgeons

**FIGURE 3:**
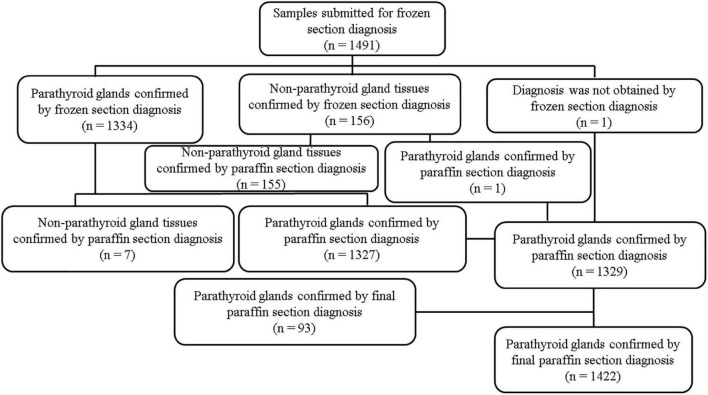
Number of samples submitted for frozen section diagnosis and pathological diagnosis. *n* = number of specimens.

The number of samples submitted for frozen section diagnosis and pathological diagnosis is summarized in Figure 3. A total of 1,491 samples were resected and submitted for frozen section diagnosis based on the surgeons’ PTG diagnosis. Out of 1,491 samples submitted, 1,334 were diagnosed as PTGs by frozen section diagnosis. Frozen section diagnosis was confirmed by paraffin section diagnosis using the same samples used for frozen section diagnosis. Among the 1,334 samples diagnosed as PTGs by frozen section diagnosis, seven samples were misdiagnosed; three thyroid and four lymph nodes were misdiagnosed as PTGs by frozen section diagnosis. Among 156 samples diagnosed as “other tissues” by frozen section diagnosis, one sample was misdiagnosed; one PTG was misdiagnosed as a lymph node by frozen section diagnosis. Out of 1,491 samples, one sample was not diagnosed by frozen section diagnosis; out of 1,490 samples, 1,482 PTGs were accurately diagnosed. The accuracy of frozen section diagnosis was 99.4% (1,482/1,490). The surgeons’ accuracy, estimated from the number of samples submitted for frozen section diagnosis with suspected PTGs, was 88.9% (1,329/1,491). Out of 162 samples that were erroneously resected and submitted by surgeons for frozen section diagnosis, 69 were thyroid glands (10.9%); 55, lymph nodes (3.7%); 24, adipose tissues (1.6%); 7, connective tissues (0.5%); and 7, thymuses (0.5%) (Table 7).

**TABLE 7:** Surgeons’ and frozen section diagnoses.

			Surgeons’ diagnosis	Frozen section diagnosis
			*n* = 1491	*n* = 1491
Correct diagnosis (%)			1329 (89.1)	1482 (99.4)
	Parathyroid gland (%)		1329 (89.1)	1327 (89.0)
	Other tissues (%)		0	155 (10.4)
		Thyroid (%)	0	66 (4.4)
		Lymph node (%)	0	51 (3.4)
		Fat tissues (%)	0	24 (1.6)
		Connective tissues (%)	0	7 (0.5)
		Thymus (%)	0	7 (0.5)
Misdiagnosis (%)			162 (10.9)	8 (0.5)
	Thyroid misdiagnosed as parathyroid (%)		69 (4.6)	3 (0.2)
	Lymph node misdiagnosed as parathyroid (%)		55 (3.7)	4 (0.3)
	Fat tissues misdiagnosed as parathyroid (%)		24 (1.6)	0
	Connective tissues misdiagnosed as parathyroid (%)		7 (0.5)	0
	Thymus misdiagnosed as parathyroid (%)		7 (0.5)	0
	Parathyroid gland misdiagnosed as lymph node (%)		0	1 (0.1)
Undiagnosed parathyroid during operation (%)			0	1 (0.1)

CI, confidence interval.

### Details of supernumerary, usual four, and infranumerary parathyroid glands

The details of the supernumerary and usual four PTGs are presented in Table 8. Among the 1,422 PTGs confirmed by final paraffin section diagnosis, 90 supernumerary PTGs (6.3%) were confirmed by final paraffin section diagnosis (Table 9). The number of supernumerary PTGs identified and unidentified by frozen section diagnosis was 21 and 69 PTGs, respectively. A total of 1,332 of the usual four PTGs were confirmed by the final paraffin section diagnosis. The number of usual four PTGs identified and unidentified by frozen section diagnosis was 1,308 and 24 PTGs, respectively. Of 1,422 PTGs, 93 (6.5%) were unidentified by frozen section diagnosis. Of 93 PTGs unidentified by frozen section diagnosis, 69 PTGs were supernumerary PTGs. Of 69 unidentified supernumerary PTGs, 64 (92.8%) were found in the thymus (Table 8). In 344 patients, 44 infranumerary PTGs were identified (Table 10).

**TABLE 8:** Localization of parathyroid glands and identification by frozen section diagnosis in all parathyroid glands confirmed by final paraffin section diagnosis.

	Supernumerary PTGs identified by frozen section diagnosis	Supernumerary PTGs unidentified by frozen section diagnosis	Usual four PTGs identified by frozen section diagnosis	Usual four PTGs unidentified by frozen section diagnosis	Total
Localization (%)	21 (1.5)	69 (4.9)	1308 (92.0)	24 (1.7)	1422
Intramediastinum PTG (%)	0	0	1 (0.1)	0	1
Intrathymus PTG (%)	1 (4.8)	64 (92.8)	223 (17.0)	8 (33.3)	296
Intrathyroid PTG (%)	1 (4.8)	3 (4.3)	13 (1.0)	2 (8.3)	19
Intracarotid arterial sheath PTG (%)	1 (4.8)	0	0	0	1
Left upper PTG (%)	2 (9.5)	1 (1.4)	338 (25.8)	1 (4.2)	342
Left lower PTG (%)	10 (47.6)	1 (1.4)	188 (14.4)	2 (8.3)	201
Right upper PTG (%)	0	0	329 (25.2)	5 (20.8)	334
Right lower PTG (%)	6 (28.6)	0	216 (16.5)	6 (25.0)	228

PTG, parathyroid gland.

**TABLE 9:** Details of supernumerary parathyroid glands.

		Patients with supernumerary PTGs
		344
Number of supernumerary PTGs	0 (%)	268 (77.9)
	1 (%)	64 (18.6)
	2 (%)	11 (3.2)
	3 (%)	0
	4 (%)	1 (0.3)

		**Number of supernumerary PTGs**
		**90**

Localization of supernumerary PTGs	Intrathymus PTG (%)	65 (72.2)
	Intrathyroid PTG (%)	4 (4.4)
	Intracarotid arterial sheath PTG (%)	1 (1.1)
	Left upper PTG (%)	3 (3.3)
	Left lower PTG (%)	11 (12.2)
	Right upper PTG (%)	0
	Right lower PTG (%)	6 (6.7)

PTG, parathyroid gland.

**TABLE 10:** Details of infranumerary parathyroid glands.

		Patients with infranumerary PTGs
		344
Number of infranumerary PTGs	0 (%)	308 (89.5)
	1 (%)	29 (8.4)
	2 (%)	6 (1.7)
	3 (%)	1 (0.3)

		**Number of infranumerary PTGs**
		**44**

Localization of infranumerary PTGs	Left upper PTG (%)	4 (9.1)
	Left lower PTG (%)	15 (34.1)
	Right upper PTG (%)	7 (15.9)
	Right lower PTG (%)	18 (40.9)

PTG, parathyroid gland.

### Preoperative localization by imaging studies

The localization of each imaging modality in all patients is shown in Supplementary Tables 2a–d. The diagnostic validity of each imaging modality in all patients is shown in Table 11. The accuracy of imaging studies by CT, US, ^99m^Tc-MIBI, and a combination of CT, US, and ^99m^Tc-MIBI was 54.1, 52.7, 38.1, and 66.8%, respectively. The localization of imaging modalities in patients who underwent successful PTx is shown in Supplementary Tables 3a–d, whereas the diagnostic validity of each imaging modality in patients who underwent successful PTx is shown in Table 12. The accuracy of imaging studies by CT, US, ^99m^Tc-MIBI, and a combination of CT, US, and ^99m^Tc-MIBI in patients who underwent successful PTx was 54.0, 52.8, 38.2, and 66.9%, respectively. None of the 90 supernumerary PTGs was identified by any preoperative imaging studies (Table 8).

**TABLE 11:** Diagnostic validity of preoperative imaging studies in all patients.

	CT	US	^99m^Tc-MIBI	CT, US, and ^99m^Tc-MIBI
Sensitivity %	53.6	52.3	37.2	67.5
Specificity %	69.4	63.5	65.2	46.0
Positive predictive value %	98.1	97.5	97.0	97.3
Negative predictive value %	4.9	4.6	3.3	4.7
Accuracy %	54.1	52.7	38.1	66.8

CT, computed tomography; ^99*m*^Tc-MIBI, technetium-99m methoxyisobutylisonitrile scintigraphy; US, ultrasonography.

**TABLE 12:** Diagnostic validity of preoperative imaging studies in patients with successful PTx.

	CT	US	^99m^Tc-MIBI	CT, US, and ^99m^Tc-MIBI
Sensitivity %	53.5	52.5	37.5	67.4
Specificity %	71.1	62.5	64.7	47.4
Positive predictive value %	98.5	97.9	97.6	97.8
Negative predictive value %	4.1	3.8	2.6	4.0
Accuracy %	54.0	52.8	38.2	66.9

CT, computed tomography; ^99*m*^Tc-MIBI, technetium-99m methoxyisobutylisonitrile scintigraphy; US, ultrasonography.

### Number of parathyroid glands unidentified by frozen section diagnosis but identified by final paraffin section diagnosis

A total of 93 PTGs were unidentified and were not submitted for frozen section diagnosis (Supplementary Table 4). Whereas four PTGs were identified by preoperative imaging studies: one right upper PTG by US; one right upper PTG by CT, US, and 99mTc-MIBI; and two right lower PTGs by US.

## Discussion

This study investigated the preoperative and intraoperative factors contributing to successful PTx. IOIPTH monitoring and the number of PTGs identified by frozen section diagnosis contributed to successful PTx. In particular, IOIPTH monitoring was useful in patients with <4 PTGs identified during PTx. On the other hand, preoperative imaging studies and the number of PTGs submitted for frozen section diagnosis by surgeons during PTx were factors not contributing to the successful PTx.

In the present study, multivariate analysis showed that IOIPTH monitoring was useful for successful PTx. The cutoff for IOIPTH levels has been extensively studied, although these studies did not simultaneously examine preoperative imaging and frozen section diagnosis (17–19, 22–25). We used a >70% decrease in intact PTH levels measured 10 min after total PTx and transcervical thymectomy to define a sufficient decrease in IOIPTH levels for successful PTx. The definition of sufficient decrease in IOIPTH levels for successful PTx was different from the definition of successful PTx, because the IOIPTH assay measures not only biologically active 1–84 PTH levels but also 7–84 PTH levels. The 7–84 PTH fragment is mainly excreted in the urine. The half-life of 7–84 PTH is several hours, although the half-life of 1–84 PTH is around 3–4 min (26). Because the duration of the operation is limited, it is difficult to complete intact PTH levels of <60 pg/mL during the operation. A decrease in intact PTH levels > 70% was defined as a sufficient decrease in IOIPTH levels for successful PTx. The rates of recurrent or persistent SHPT were compared between groups with intact PTH levels of <60 pg/mL on POD 1 and intact PTH levels of ≥ 60 pg/mL on POD 1 using Cox regression analysis adjusted for differences in patient characteristics with IPTW. A significantly lower recurrence or persistence rate of SHPT in the group with intact PTH levels < 60 pg/mL on POD 1 was identified. For PTx to be considered successful, intact PTH levels of <60 pg/mL on POD 1 were required. The difficulty of PTx for SHPT is the unexpected presence of supernumerary and infranumerary PTGs. In PTx for SHPT, complete removal of PTGs is essential to prevent recurrent and persistent SHPT because remnant PTGs in chronic kidney disease can be stimulated and secrete excess intact PTH (15, 16). Additional PTx may be a risk factor for recurrent laryngeal nerve injury due to adhesions after the initial PTx. In this study, 90 supernumerary PTGs were identified by final paraffin section diagnosis. Out of 90 supernumerary PTGs, 69 were not identified by frozen section diagnosis. Even in patients who underwent successful PTx, 18 infranumerary PTGs were identified. A total of 93 out of 1422 PTGs confirmed by final paraffin section diagnosis were not identified by surgeons during PTx. Of the 93 PTGs, 89 (95.7%) were not identified by preoperative imaging studies. These facts imply that preoperative imaging studies and surgeons’ identification of PTGs during surgery is insufficient for successful PTx. Accordingly, IOIPTH monitoring is useful for successful PTx. If a sufficient decrease in IOIPTH is not completed even after removing the usual four PTGs, it indicates the exploration of supernumerary PTGs. Additionally, IOIPTH monitoring proved to be useful in patients with <4 PTGs identified during surgery in this study, although the analysis was performed using univariate analysis. In previous studies, patients with <4 identified PTGs accounted for 2–6% of the general population (21, 27, 28). If four PTGs are unidentified, the decision to end surgery may be difficult, and excessive exploration may result in an unexpected injury to the recurrent laryngeal nerve. In such a case, IOIPTH monitoring may indicate the need for further exploration or the completion of surgery.

In this present study, the diagnostic accuracies of imaging studies were investigated. The diagnostic accuracy of localization of PTGs by the combination of CT, US, and ^99m^Tc-MIBI was the highest. Similarly, in the previous report, the diagnostic accuracy of localization was the highest when diagnosed by the combination of CT, US, and ^99m^Tc-MIBI (75.1%), because the diagnostic accuracy of ectopic PTGs was higher in the combination of CT, US, and ^99m^Tc-MIBI than that in any of the single imaging modality (21). However, in this study, the number of PTGs identified by preoperative imaging studies did not affect successful PTx, because none of 90 supernumerary PTGs (6.3%) might be identified by preoperative imaging studies in the present study. Similarly, in previous reports, the identification rate of supernumerary PTGs by preoperative imaging studies was low, although the incidence of supernumerary PTGs in patients with SHPT was 5–30% (21, 29–31). In contrast, in the present study, 21 out of 90 supernumerary PTGs were identified by surgeons. This suggests that the surgeons’ diagnosis during PTx for supernumerary PTGs may be more reliable than that by preoperative imaging studies. However, the number of samples submitted for frozen section diagnosis, which might be the surgeons’ diagnosis during PTx, was not an independent factor for successful PTx. This result could be due to the insufficient diagnostic accuracy of surgeons. The main causes of misdiagnosis by surgeons were misdiagnosis of the thyroid, lymph nodes, and fat tissues as PTGs. On the other hand, the number of PTGs identified by frozen section diagnosis was independent factors for successful PTx in the multivariate regression analysis. This may be because this study’s diagnostic accuracy of PTGs by frozen section diagnosis was quite high (99.4%). This result was similar to that in a previous study (99.2%) (32). On frozen section diagnosis, thyroid and lymph nodes were misdiagnosed as PTGs in only 3 and 4 samples, respectively. Only one PTG was misdiagnosed as a lymph node by frozen section diagnosis. This suggests that frozen section diagnosis may be necessary for PTx for SHPT.

The limitations of this study were its retrospective nature and lack of data on the preoperative medical treatment for SHPT. Future prospective studies are needed to investigate the effects of frozen section diagnosis and IOIPTH monitoring in PTx for SHPT for successful PTx, including preoperative and intraoperative factors. However, in this study, the importance of frozen section diagnosis and IOIPTH monitoring in PTx for SHPT was demonstrated in the multivariate analysis. The details of the results of imaging studies, supernumerary and usual four PTGs, and misdiagnoses in the frozen section and surgeons’ diagnosis were revealed.

In conclusion, frozen section diagnosis and IOIPTH monitoring are necessary to achieve successful PTx for SHPT.

## Data availability statement

The raw data supporting the conclusions of this article will be made available by the authors, without undue reservation.

## Ethics statement

The studies involving human participants were reviewed and approved by Institutional Review Board of the Japanese Red Cross Aichi Medical Center Nagoya Daini Hospital. Written informed consent for participation was not required for this study in accordance with the national legislation and the institutional requirements.

## Author contributions

TH designed and acquired the data, interpreted the results, and drafted the manuscript. MO acquired the data. YH, KF, NG, YT, SN, YW, and TI interpreted the results. All authors approved the final version of the manuscript.

## Abbreviations

SHPT: secondary hyperparathyroidism
POD: postoperative day
PTx: parathyroidectomy
PTG: parathyroid gland
PTH: parathyroid hormone
IOIPTH: intraoperative intact PTH
OR: odds ratio
CI: confidence interval
ESRD: end-stage renal disease
STROBE: strengthening the reporting of observational studies in epidemiology
IPTW: inverse probability of treatment weighting
CKD-MBD: chronic kidney disease-related mineral and bone disorder
CT: computed tomography
US: ultrasonography
^99*m*^Tc-MIBI: technetium-99m methoxyisobutylisonitrile scintigraphy.

## References

[1] Lau WL Obi Y Kalantar-Zadeh K. Parathyroidectomy in the management of secondary hyperparathyroidism. *Clin J Am Soc Nephrol.* (2018) 13:952–61. 10.2215/CJN.10390917 29523679 PMC5989682

[2] Block GA Klassen PS Lazarus JM Ofsthun N Lowrie EG Chertow GM. Mineral metabolism, mortality, and morbidity in maintenance hemodialysis. *J Am Soc Nephrol.* (2004) 15:2208–18. 10.1097/01.ASN.0000133041.27682.A2 15284307

[3] Tentori F Blayney MJ Albert JM Gillespie BW Kerr PG Bommer J Mortality risk for dialysis patients with different levels of serum calcium, phosphorus, and PTH: the dialysis outcomes and practice patterns study (DOPPS). *Am J Kidney Dis.* (2008) 52:519–30. 10.1053/j.ajkd.2008.03.020 18514987

[4] Taniguchi M Fukagawa M Fujii N Hamano T Shoji T Yokoyama K Serum phosphate and calcium should be primarily and consistently controlled in prevalent hemodialysis patients. *Ther Apher Dial.* (2013) 17:221–8. 10.1111/1744-9987.12030 23551679

[5] Tominaga Y Kakuta T Yasunaga C Nakamura M Kadokura Y Tahara H. Evaluation of parathyroidectomy for secondary and tertiary hyperparathyroidism by the parathyroid surgeons’ society of Japan. *Ther Apher Dial.* (2016) 20:6–11. 10.1111/1744-9987.12352 26879490

[6] Komaba H Nakanishi S Fujimori A Tanaka M Shin J Shibuya K Cinacalcet effectively reduces parathyroid hormone secretion and gland volume regardless of pretreatment gland size in patients with secondary hyperparathyroidism. *Clin J Am Soc Nephrol.* (2010) 5:2305–14. 10.2215/CJN.02110310 20798251 PMC2994093

[7] Ichii M Ishimura E Okuno S Chou H Kato Y Tsuboniwa N Decreases in parathyroid gland volume after cinacalcet treatment in hemodialysis patients with secondary hyperparathyroidism. *Nephron Clin Pract.* (2010) 115:c195–202. 10.1159/000313035 20413997

[8] Block GA Martin KJ de Francisco AL Turner SA Avram MM Suranyi MG Cinacalcet for secondary hyperparathyroidism in patients receiving hemodialysis. *N Engl J Med.* (2004) 350:1516–25. 10.1056/NEJMoa031633 15071126

[9] Komaba H Kakuta T Fukagawa M. Management of secondary hyperparathyroidism: how and why? *Clin Exp Nephrol.* (2017) 21:37–45. 10.1007/s10157-016-1369-2 28044233

[10] Fukagawa M Yokoyama K Koiwa F Taniguchi M Shoji T Kazama JJ Clinical practice guideline for the management of chronic kidney disease-mineral and bone disorder. *Ther Apher Dial.* (2013) 17:247–88. 10.1111/1744-9987.12058 23735142

[11] Kim SM Long J Montez-Rath ME Leonard MB Norton JA Chertow GM. Rates and outcomes of parathyroidectomy for secondary hyperparathyroidism in the United States. *Clin J Am Soc Nephrol.* (2016) 11:1260–7. 10.2215/CJN.10370915 27269300 PMC4934842

[12] Yamamoto M Ogata H Mizobuchi M Yoshida N Kumata-Maeta C Koiwa F Number of enlarged parathyroid glands might be a predictor of Cinacalcet response in advanced secondary hyperparathyroidism. *Clin Exp Nephrol.* (2012) 16:292–9. 10.1007/s10157-011-0547-5 22011886

[13] Hong YA Cho YS Kim SW Jung MY Lee EA Ko GJ Diameter of parathyroid glands measured by computed tomography as a predictive indicator for response to cinacalcet in dialysis patients with secondary hyperparathyroidism. *Kidney Blood Press Res.* (2015) 40:277–87. 10.1159/000368503 26022985

[14] Hou J Shan H Zhang Y Deng X Guo B Kang J Network meta-analysis of surgical treatment for secondary hyperparathyroidism. *Am J Otolaryngol.* (2020) 41:102370. 10.1016/j.amjoto.2019.102370 31889554

[15] Hibi Y Tominaga Y Sato T Katayama A Haba T Uchida K Reoperation for renal hyperparathyroidism. *World J Surg.* (2002) 26:1301–7. 10.1007/s00268-002-6731-8 12205559

[16] Tominaga Y Katayama A Sato T Matsuoka S Goto N Haba T Re-operation is frequently required when parathyroid glands remain after initial parathyroidectomy for advanced secondary hyperparathyroidism in uraemic patients. *Nephrol Dial Transplant.* (2003) 18(Suppl 3.):iii65–70. 10.1093/ndt/gfg1017 12771305

[17] Chávez KV Márquez-González H Chavez-Tostado M. The usefulness of intraoperative PTH as a predictor for successful parathyroidectomy in secondary hyperparathyroidism. *Front Surg.* (2021) 8:696469. 10.3389/fsurg.2021.696469 34262935 PMC8273272

[18] Silveira AA Brescia MDG do Nascimento CP Jr Arap SS Montenegro FLM. Critical analysis of the intraoperative parathyroid hormone decrease during parathyroidectomy for secondary and tertiary hyperparathyroidism. *Surgery.* (2020) 168:1079–85. 10.1016/j.surg.2020.06.043 32811697

[19] Moor JW Roberts S Atkin SL England RJA. Intraoperative parathyroid hormone monitoring to determine long-term success of total parathyroidectomy for secondary hyperparathyroidism. *Head Neck.* (2011) 33:293–6. 10.1002/hed.21441 20848450

[20] Fuster D Ybarra J Ortin J Torregrosa JV Gilabert R Setoain X Role of pre-operative imaging using 99mTc-MIBI and neck ultrasound in patients with secondary hyperparathyroidism who are candidates for subtotal parathyroidectomy. *Eur J Nucl Med Mol Imaging.* (2006) 33:467–73. 10.1007/s00259-005-0021-2 16404597

[21] Hiramitsu T Tomosugi T Okada M Futamura K Tsujita M Goto N Pre-operative localisation of the parathyroid glands in secondary hyperparathyroidism: a retrospective cohort study [Sci. rep.]. *Sci Rep.* (2019) 9:14634. 10.1038/s41598-019-51265-y 31602011 PMC6787184

[22] Hiramitsu T Tominaga Y Okada M Yamamoto T Kobayashi T. A retrospective study of the impact of intraoperative intact parathyroid hormone monitoring during total parathyroidectomy for secondary hyperparathyroidism: stard study. *Medicine.* (2015) 94:e1213. 10.1097/MD.0000000000001213 26200645 PMC4603015

[23] Lee JB Kim WY Lee Y. The role of pre-operative ultrasonography, computed tomography, and sestamibi scintigraphy localization in secondary hyperparathyroidism. *Ann Surg Treat Res.* (2015) 89:300–5. 10.4174/astr.2015.89.6.300 26665124 PMC4672093

[24] Zhang L Xing C Shen C Zeng M Yang G Mao H Diagnostic accuracy study of intraoperative and perioperative serum intact PTH level for successful parathyroidectomy in 501 secondary hyperparathyroidism patients. *Sci Rep.* (2016) 6:26841. 10.1038/srep26841 27231027 PMC4882599

[25] Steinl GK Kuo JH. Surgical management of secondary hyperparathyroidism. *Kidney Int Rep.* (2021) 6:254–64. 10.1016/j.ekir.2020.11.023 33615051 PMC7879113

[26] Yajima A Tsuchiya K Kuro-o M Urena P Tominaga Y Okada M Renal hyperparathyroidism. *Vitam Horm.* (2022) 120:305–43. 10.1016/bs.vh.2022.04.010 35953115

[27] Hibi Y Tominaga Y Uchida K Takagi H Imai T Funahashi H Cases with fewer than four parathyroid glands in patients with renal hyperparathyroidism at initial parathyroidectomy. *World J Surg.* (2002) 26:314–7. 10.1007/s00268-001-0224-z 11865367

[28] Randolph GW. *Surgery of the Thyroid and Parathyroid Glands.* 3rd ed. Philadelphia: Elsevier – Health Sciences Division (2020). p. 15–25.

[29] Vulpio C Bossola M De Gaetano A Maresca G Bruno I Fadda G Usefulness of the combination of ultrasonography and 99mTc-sestamibi scintigraphy in the pre-operative evaluation of uremic secondary hyperparathyroidism. *Head Neck.* (2010) 32:1226–35. 10.1002/hed.21320 20091692

[30] Reitz RJ III Dreimiller A Khil A Horwitz E McHenry CR. Ectopic and supernumerary parathyroid glands in patients with refractory renal hyperparathyroidism. *Surgery.* (2021) 169:513–8. 10.1016/j.surg.2020.08.007 32919783

[31] Pattou FN Pellissier LC Noël C Wambergue F Huglo DG Proye CA. Supernumerary parathyroid glands: frequency and surgical significance in treatment of renal hyperparathyroidism. *World J Surg.* (2000) 24:1330–4. 10.1007/s002680010220 11038202

[32] Westra WH Pritchett DD Udelsman R. Intraoperative confirmation of parathyroid tissue during parathyroid exploration: a retrospective evaluation of the frozen section. *Am J Surg Pathol.* (1998) 22:538–44. 10.1097/00000478-199805000-00003 9591722

