# Supplementary material for: Intraoperative intact parathyroid hormone monitoring and frozen section diagnosis are essential for successful parathyroidectomy in secondary hyperparathyroidism
Source: Front Med (Lausanne). 2022 Nov 7;9:1007887. doi: 10.3389/fmed.2022.1007887 (PMC9676982; doi:10.3389/fmed.2022.1007887)
Supplement: Supplementary file 1 [file Data_Sheet_1.docx]

Supplementary Material

# Supplementary Table 1

| Supplementary Table 1. Patient characteristics with <4 parathyroid glands identified by frozen section diagnosis | | | | | | |
| --- | --- | --- | --- | --- | --- | --- |
|  | **Unsuccessful PTx group** | **Successful PTx group** | ***P* value** | **Odds ratio** | **95% CI** | |
|  | ***n* = 20** | ***n* = 30** |  |  |  |  |
| Sex male (%) | 14 (70.0) | 19 (63.3) | 0.763 | 0.740 | 0.221 | 2.484 |
| Age years (SD) | 54.3 (12.9) | 55.8 (11.0) | 0.960 |  |  |  |
| Height cm (SD) | 161.2 (8.2) | 163.1 (10.1) | 0.357 |  |  |  |
| Body weight kg (SD) | 53.1 (5.6) | 61.3 (15.4) | 0.327 |  |  |  |
| Body mass index kg/m^2^ (SD) | 20.4 (1.4) | 22.8 (4.2) | 0.501 |  |  |  |
| Dialysis vintage months (SD) | 179.5 (98.7) | 154.0 (84.3) | 0.411 |  |  |  |
| Preoperative serum albumin level g/dL (SD) | 4.02 (0.34) | 3.93 (0.42) | 0.620 |  |  |  |
| Preoperative serum alkaline phosphatase level U/L (SD) | 287.5 (106.2) | 327.9 (173.5) | 0.513 |  |  |  |
| Preoperative serum calcium level mg/dL (SD) | 9.7 (0.8) | 9.6 (0.8) | 0.613 |  |  |  |
| Preoperative serum calcium level corrected by serum albumin level mg/dL (SD) | 9.8 (0.7) | 9.8 (0.7) | 0.452 |  |  |  |
| Preoperative serum phosphorus level mg/dL (SD) | 5.9 (1.3) | 6.2 (1.6) | 0.482 |  |  |  |
| Preoperative CT (%) | 20 (100.0) | 30 (100.0) | NA |  |  |  |
| Preoperative US (%) | 20 (100.0) | 30 (100.0) | NA |  |  |  |
| Preoperative ^99m^Tc-MIBI (%) | 19 (95.0) | 30 (100.0) | 0.400 |  |  |  |
| Number of PTGs identified by preoperative CT (SD) | 2.1 (1.1) | 1.7 (0.9) | 0.192 |  |  |  |
| Number of PTGs identified by preoperative US (SD) | 2.1 (1.2) | 2.0 (1.1) | 0.829 |  |  |  |
| Number of PTGs identified by preoperative ^99m^Tc-MIBI (SD) | 1.7 (1.1) | 1.3 (0.8) | 0.224 |  |  |  |
| Number of PTGs identified by preoperative CT, US, and ^99m^Tc-MIBI (SD) | 2.8 (1.1) | 2.3 (1.1) | 0.151 |  |  |  |
| Intact PTH levels at admission pg/mL (SD) | 453.0 (285.7) | 635.1 (565.0) | **0.023** |  |  |  |
| Number of samples submitted for frozen section diagnosis (SD) | 3.7 (0.7) | 4.0 (1.3) | 0.247 |  |  |  |
| Number of PTGs identified by frozen section diagnosis (SD) | 2.7 (0.5) | 2.7 (0.6) | **0.025** |  |  |  |
| Number of PTGs identified by final paraffin section diagnosis (SD) | 2.8 (0.9) | 3.7 (0.8) | **0.001** |  |  |  |
| Intraoperative intact PTH levels before skin incision ng/mL (SD) | 500.2 (302.8) | 620.0 (429.3) | 0.025 |  |  |  |
| Intraoperative intact PTH levels at 10 min after total PTx and transcervical thymectomy pg/mL (SD) | 308.4 (288.1) | 78.1 (73.0) | **< 0.001** |  |  |  |
| Decrease in intraoperative intact PTH levels % (SD) | 43.2 (37.7) | 87.7 (5.8) | **<0.001** |  |  |  |
| Decrease in intraoperative intact PTH levels >70% (%) | 11 (55.0) | 29 (96.7) | **0.001** | 23.727 | 2.684 | 209.782 |
| Intact PTH levels on POD 1 pg/mL (SD) | 149.8 (111.3) | 52.9 (87.5) | **<0.001** |  |  |  |
| Persistent and recurrent SHPT in the neck or mediastinum during the observation period (%) | 3 (15.0) | 2 (6.7) | 0.377 | 0.405 | 0.061 | 2.674 |
| Observation period months (SD) | 85.7 (33.7) | 88.7 (33.4) | 0.751 |  |  |  |

| CI, confidence interval; CT, computed tomography; POD, postoperative day; PTGs, parathyroid glands; PTH, parathyroid hormone; PTx, parathyroidectomy; SD, standard deviation; 99mTc-MIBI, technetium-99m methoxyisobutylisonitrile scintigraphy; US, ultrasonography. Boldface indicates statistically significant results. |
| --- |

# Supplementary Table 2

| **Supplementary Table 2a**. Preoperative localization diagnosis by computed tomography | | | |
| --- | --- | --- | --- |
|  | **Resected PTGs** | **Non-resected PTGs** |  |
| Identified PTGs in CT | 762 | 15 | 777 |
| Non-identified PTGs in CT | 660 | 34 | 694 |
|  | 1422 | 49 | 1471 |
| CT, computed tomography; PTG, parathyroid gland. | | | |
|  |  |  |  |
| **Supplementary Table 2b**. Preoperative localization diagnosis by ultrasonography | | | |
|  | Resected PTGs | **Non-resected PTGs** |  |
| Identified PTGs in US | 744 | 19 | 763 |
| Non-identified PTGs in US | 678 | 33 | 711 |
|  | 1422 | 52 | 1474 |
| PTG, parathyroid gland; US, ultrasonography. | | | |
|  |  |  |  |
| **Supplementary Table 2c**. Preoperative localization diagnosis by ^99m^Tc-MIBI scintigraphy | | | |
|  | **Resected PTGs** | **Non-resected PTGs** |  |
| Identified PTGs in ^99m^Tc-MIBI | 518 | 16 | 534 |
| Non-identified PTGs in ^99m^Tc-MIBI | 873 | 30 | 903 |
|  | 1391 | 46 | 1437 |
| PTG, parathyroid gland; ^99m^Tc-MIBI, technetium-99m methoxyisobutylisonitrile scintigraphy. | | | |
| **Supplementary Table 2d**. Preoperative localization diagnosis by computed tomography, ultrasonography, and ^99m^Tc-MIBI scintigraphy | | | |
|  | **Resected PTGs** | **Non-resected PTGs** |  |
| Identified PTGs in CT, US, and ^99m^Tc-MIBI | 960 | 27 | 987 |
| Non-identified PTGs in CT, US, and ^99m^Tc-MIBI | 462 | 23 | 485 |
|  | 1422 | 50 | 1472 |
| CT, computed tomography; PTGs, parathyroid glands; ^99m^Tc-MIBI, technetium-99m methoxyisobutylisonitrile scintigraphy; US, ultrasonography. Supplementary Table 3  \| **Supplementary Table 3a**. Preoperative localization diagnosis by computed tomography \| \| \| \| \| --- \| --- \| --- \| --- \| \|  \| **Resected PTGs** \| **Non-resected PTGs** \|  \| \| Identified PTGs in CT \| 717 \| 11 \| 728 \| \| Non-identified PTGs in CT \| 624 \| 27 \| 651 \| \|  \| 1341 \| 38 \| 1379 \| \| CT, computed tomography; PTGs, parathyroid glands. \| \| \| \| \|  \|  \|  \|  \| \| **Supplementary Table 3b**. Preoperative localization diagnosis by ultrasonography \| \| \| \| \|  \| **Resected PTGs** \| **Non-resected PTGs** \|  \| \| Identified PTGs in US \| 704 \| 15 \| 719 \| \| Non-identified PTGs in US \| 637 \| 25 \| 662 \| \|  \| 1341 \| 40 \| 1381 \| \| PTGs, parathyroid glands; US, ultrasonography. \| \| \| \| \|  \|  \|  \|  \| \| **Supplementary Table 3c**. Preoperative localization diagnosis by ^99m^Tc-MIBI scintigraphy \| \| \| \| \|  \| **Resected PTGs** \| **Non-resected PTGs** \|  \| \| Identified PTGs in ^99m^Tc-MIBI \| 491 \| 12 \| 503 \| \| Non-identified PTGs in ^99m^Tc-MIBI \| 819 \| 22 \| 841 \| \|  \| 1310 \| 34 \| 1344 \| \| PTGs, parathyroid glands; ^99m^Tc-MIBI, technetium-99m methoxyisobutylisonitrile scintigraphy. \| \| \| \| \|  \|  \|  \|  \| \| **Supplementary Table 3d**. Preoperative localization diagnosis by computed tomography, ultrasonography, and ^99m^Tc-MIBI scintigraphy \| \| \| \| \|  \| **Resected PTGs** \| **Non-resected PTGs** \|  \| \| Identified PTGs in CT, US, and ^99m^Tc-MIBI \| 904 \| 20 \| 924 \| \| Non-identified PTGs in CT, US, and ^99m^Tc-MIBI \| 437 \| 18 \| 455 \| \|  \| 1341 \| 38 \| 1379 \| \| CT, computed tomography; PTGs, parathyroid glands; ^99m^Tc-MIBI, technetium-99m methoxyisobutylisonitrile scintigraphy; US, ultrasonography. Supplementary Table 4  \| **Supplementary Table 4**. Number of parathyroid glands identified by final paraffin section diagnosis in samples not submitted for frozen section diagnosis \| \| \| \| \| --- \| --- \| --- \| --- \| \| **Localization** \| **Number of PTGs** \| **Number of identified PTGs by preoperative imaging studies** \| **Modality of imaging studies** \| \| Left upper PTG \| 2 \| 0 \|  \| \| Left lower PTG \| 3 \| 0 \|  \| \| Right upper PTG \| 5 \| 2 \| 1: US, 1: CT, US, and ^99m^Tc-MIBI \| \| Right lower PTG \| 6 \| 2 \| 2: US \| \| Intrathyroid PTG \| 5 \| 0 \|  \| \| Intrathymus PTG \| 72 \| 0 \|  \| \| CT, computed tomography; US, ultrasonography; PTG, parathyroid gland; ^99m^Tc-MIBI, technetium-99m methoxyisobutylisonitrile scintigraphy \| \| \| \| \| \| \| \| | | | |
